# Weakly Supervised Deep Learning for Tooth-Marked Tongue Recognition

**DOI:** 10.3389/fphys.2022.847267

**Published:** 2022-04-12

**Authors:** Jianguo Zhou, Shangxuan Li, Xuesong Wang, Zizhu Yang, Xinyuan Hou, Wei Lai, Shifeng Zhao, Qingqiong Deng, Wu Zhou

**Affiliations:** ^1^ School of Medical Information Engineering, Guangzhou University of Chinese Medicine, Guangzhou, China; ^2^ School of Artificial Intelligence, Beijing Normal University, Beijing, China; ^3^ Beijing Yikang Medical Technology Co., Ltd., Beijing, China

**Keywords:** traditional Chinese medicine, tooth-marked tongue, deep learning, weakly supervised learning, tongue diagnosis, convolutional neural network

## Abstract

The recognition of tooth-marked tongues has important value for clinical diagnosis of traditional Chinese medicine. Tooth-marked tongue is often related to spleen deficiency, cold dampness, sputum, effusion, and blood stasis. The clinical manifestations of patients with tooth-marked tongue include loss of appetite, borborygmus, gastric distention, and loose stool. Traditional clinical tooth-marked tongue recognition is conducted subjectively based on the doctor’s visual observation, and its performance is affected by the doctor’s subjectivity, experience, and environmental lighting changes. In addition, the tooth marks typically have various shapes and colors on the tongue, which make it very challenging for doctors to identify tooth marks. The existing methods based on deep learning have made great progress for tooth-marked tongue recognition, but there are still shortcomings such as requiring a large amount of manual labeling of tooth marks, inability to detect and locate the tooth marks, and not conducive to clinical diagnosis and interpretation. In this study, we propose an end-to-end deep neural network for tooth-marked tongue recognition based on weakly supervised learning. Note that the deep neural network only requires image-level annotations of tooth-marked or non-tooth marked tongues. In this method, a deep neural network is trained to classify tooth-marked tongues with the image-level annotations. Then, a weakly supervised tooth-mark detection network (WSTDN) as an architecture variant of the pre-trained deep neural network is proposed for the tooth-marked region detection. Finally, the WSTDN is re-trained and fine-tuned using only the image-level annotations to simultaneously realize the classification of the tooth-marked tongue and the positioning of the tooth-marked region. Experimental results of clinical tongue images demonstrate the superiority of the proposed method compared with previously reported deep learning methods for tooth-marked tongue recognition. The proposed tooth-marked tongue recognition model may provide important syndrome diagnosis and efficacy evaluation methods, and contribute to the understanding of ethnopharmacological mechanisms.

## 1 Introduction

**FIGURE 1:**
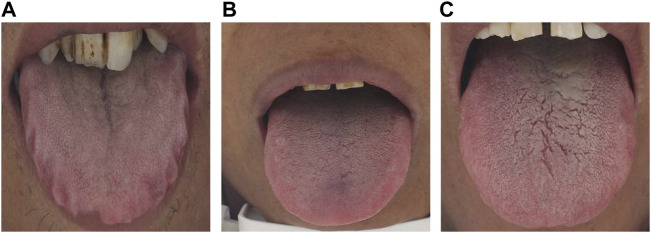
Representative tongue images. **(A)** Tooth-marked tongue with a very obvious contour distortion along both sides of the tongue, accompanied by the color change of the extruded area of the tongue; **(B)** Non-tooth marked tongue. The tongue body is flat, theperipheral contour is regular, and there is no contour distortion and color change area; **(C)** Suspicious tooth-marked tongue. The identification is controversial because the tongue body is flat and the peripheral contour is not distorted. Finally, the tooth-marked tongue is determined by the color change of the extruded area of the tongue edge.

Tongue diagnosis is one of the most important diagnostic methods in Chinese medicine. The characteristics of a tongue, such as shape and color, can reflect the internal health of the body, and the severity or progression of the disease. By observing the characteristics of a tongue, Chinese medicine can distinguish the clinical symptoms and choose appropriate treatment strategies (Zhang et al., 2017). As one of the most important tongue features, tooth marks are generally formed by the compression of the fatter tongue by adjacent teeth. Figure 1 shows some representative tooth-marked and non-tooth marked tongue images. Typically, tooth-marked tongue refers to a kind of abnormal tongue shape in which the tongue body is fat in different degrees and is compressed by the teeth, and the edge of the tongue body is formed with tooth marks that are serrated. Non-tooth-marked tongue tends to be moderately fat and thin, and the edges of the tongue are continuous and smooth. According to the theory of traditional Chinese medicine, tooth-marked tongue is often related to spleen deficiency, cold dampness, sputum, effusion, and blood stasis. The clinical manifestations of patients with tooth-marked tongue include loss of appetite, borborygmus, gastric distention, and loose stool (Li and Dong, 2017). Hence, the recognition of tooth-marked tongues has important value for clinical diagnosis of Chinese medicine. However, the routine clinical recognition of tooth-marked tongues is through the doctor’s visual observation, and its performance is limited by the doctor’s subjectivity and experience, in addition to environmental lighting changes. In addition, there are different types of tooth marks, including different colors and varied shapes, which make it challenging for doctors to identify. Therefore, the study of objective tooth-marked tongue recognition based on image data has important clinical value.

In recent years, researchers have been trying to establish an objective tooth-marked tongue recognition model based on digital image processing and analysis. Most studies are based on the local color and unevenness of the tooth scar area. Hsu et al. (2010) analyzed the RGB color composition based on the image of the tongue region and found that the G chromatogram of the tooth marks is lower than that of the tongue body and tongue surface. Lo et al. (2012) found that different imaging angles or the degree of tongue extrusion would affect the judgment of tooth marks. Li et al. (2019) used concavity information to generate suspicious tooth-marked areas for the following classification of the tooth-marked tongue. However, due to the great color difference of tongues and varied shape of tooth marks, the recognition based on color and shape of tooth marks usually has low robustness and stability.

With the continuous development of artificial intelligence and deep learning, the convolutional neural network (CNN) model is applied to tongue analysis. Xu et al. (2020) used multi-task learning with deep learning to realize tongue segmentation and tongue coating classification, and achieved better results than single task. Jiang et al. (2021) proposed toassess the tongue image quality based on a deep CNN. Hu et al. (2021) proposed a method for automatic construction of Chinese herbal prescriptions from tongue images using CNN and auxiliary latent therapy topics. Typically, current identification of the tooth-marked tongue generally extracts the entire tongue image, and then directly classifies the tooth-marked tongue based on CNN (Sun et al., 2019; Wang et al., 2020). Specifically, Sun et al. (2019) proposed a 7-layer CNN model that takes the tongue image as the input to identify the tooth-marked tongue, with an accuracy of 78.6%. Wang et al. (2020) classified the tooth-marked tongue based on a deeper CNN network, and showed that their method could achieve promising results of the tooth-marked tongue recognition. However, since the center, tip, and base of the tongue are not informative for the identification of the tooth-marked tongue, incorporating these non-informative areas into the deep neural network for analysis may have a negative impact on the performance of the model (Chong Wang et al., 2015). In addition, such tooth-marked tongue classification only provides the image-level identification of the tooth-marked tongues, and does not provide the specific location of the tooth marks, which is not conducive to assisting clinical diagnosis and interpretability.

Since tooth marks are one of the symptoms of a tongue, the identification of tooth-marked tongues has been regarded as a fine-grained classification problem, and the classification of tooth-marked tongue can be conducted by multiple instance learning (Li et al., 2019). Specifically, multiple candidates of tooth-marked areas are generated on the tongue body. If all candidates are non-tooth marks, it is a non-tooth marked tongue. On the contrary, if one candidate on the tongue is a tooth mark, it is considered as a tooth-marked tongue. Li et al. (2019) first used concavity information to generate candidates of tooth-marked areas, followed by extracting CNN deep features from these areas, and finally classified the features based on a multi-instance support vector machine (MiSVM) to classify the tooth-marked tongue. Although such pioneer work has obtained promising results for the tooth-marked tongue recognition, it has several shortcomings. First, it uses concavity information to generate candidate areas of the tongue, non-tooth marked tongues rarely have such concavity information, which makes it difficult to achieve a unified generation of candidate examples of tooth-marked and non-tooth marked tongues. Then, it requires a large amount of tooth mark examples for feature extraction, which will bring a lot of labor costs. Furthermore, deep features are extracted through the CNN model for each candidate tooth-marked region, which will also bring a lot of computational cost. Finally, this process is not an end-to-end deep neural network and cannot provide a discriminative location of the tooth-marked area, which is not conducive to assisting clinical diagnosis and interpretability. Recently, Tang et al. (2020) proposed a tongue region detection and tongue landmark detection *via* deep learning for tooth-marked tongue recognition. However, it requires a lot of image annotation including tongue landmark annotation and tongue region annotation, which is a huge burden and tedious work for clinic. Weng et al. (2021) proposed a weakly supervised tooth-mark detection method using the YOLO object detection model. However, it requires fully bounding-box level annotation of tooth marks in addition to coarse image-level annotation of tooth-marked tongue images. These are very tedious work for clinical application.

In this study, a weakly supervised object detection using deep learning is proposed for the tooth-marked tongue recognition, where only image-level labels are used for model training. The proposed method is motivated by the work of weakly supervised deep detection network in computer vision (Bilen and Vedaldi, 2016), in which a CNN pre-trained for image classification on a large dataset ImageNet is modified to reason efficiently about regions, branching off a recognition, and a detection data streams. The resulting architecture can be fine-tuned on a target dataset to achieve state-of-the-art weakly supervised object detection using only image-level annotations. Based on this consideration, the tooth-marked tongue recognition can be naturally solved from the perspective of weakly supervised object detection for two reasons. First, the detection and localization of tooth-marked areas are conducive to assisting clinical diagnosis and interpretability. Second, only image-level annotation without tooth marks labeling can significantly reduce the cost of data annotation. In addition, Zhou et al. proposed that even when there is no supervision of the target position, the convolution unit of the convolution layer can be regarded as the target detector (Zhou et al., 2015). Therefore, the classification network of tooth-marked tongues with only image-level annotations makes it possible to locate tooth marks without providing tooth mark annotations.

To this end, we propose an end-to-end deep neural network for tooth-marked tongue recognition based on weakly supervised learning. To improve the efficiency and reliability of the generation of the candidate tooth-marked areas, we use the prior knowledge of the tooth marks distribution to generate the candidate tooth-marked areas through the position information. In addition, to avoid the labeling of a large number of examples of tooth marks for deep learning, we propose a weakly supervised learning method for tooth-marked tongue recognition. Specifically, we first train a deep neural network model to classify tooth-marked tongues with the image-level annotations, and then we propose a weakly supervised tooth-mark detection network (WSTDN) as an architecture variant of the pre-trained deep neural network for the tooth-marked region detection, followed by fine-tuning the WSTDN once again using only the image-level annotations to simultaneously realize the classification of the tooth-marked tongue and the positioning of the tooth-marked region.

Compared to the existing works, the main contributions of the present work are summarized as follows: 1) we propose an end-to-end deep neural network for tooth-marked tongue recognition based on weakly supervised learning, avoiding manual labeling, and screening of a large number of tooth-marked examples; 2) we propose a novel method for generating candidate regions based on prior knowledge of tooth mark distribution to improve the efficiency of tongue tooth-marked candidate region generation; 3) in the case of only image-level labels, we propose the WSTDN to realize the classification of the tooth-marked tongue and the positioning of the tooth-marked area at the same time, which is convenient for assisting clinical diagnosis and interpretation.

## 2 Materials and Methods

### 2.1 Clinical Data

**FIGURE 2:**
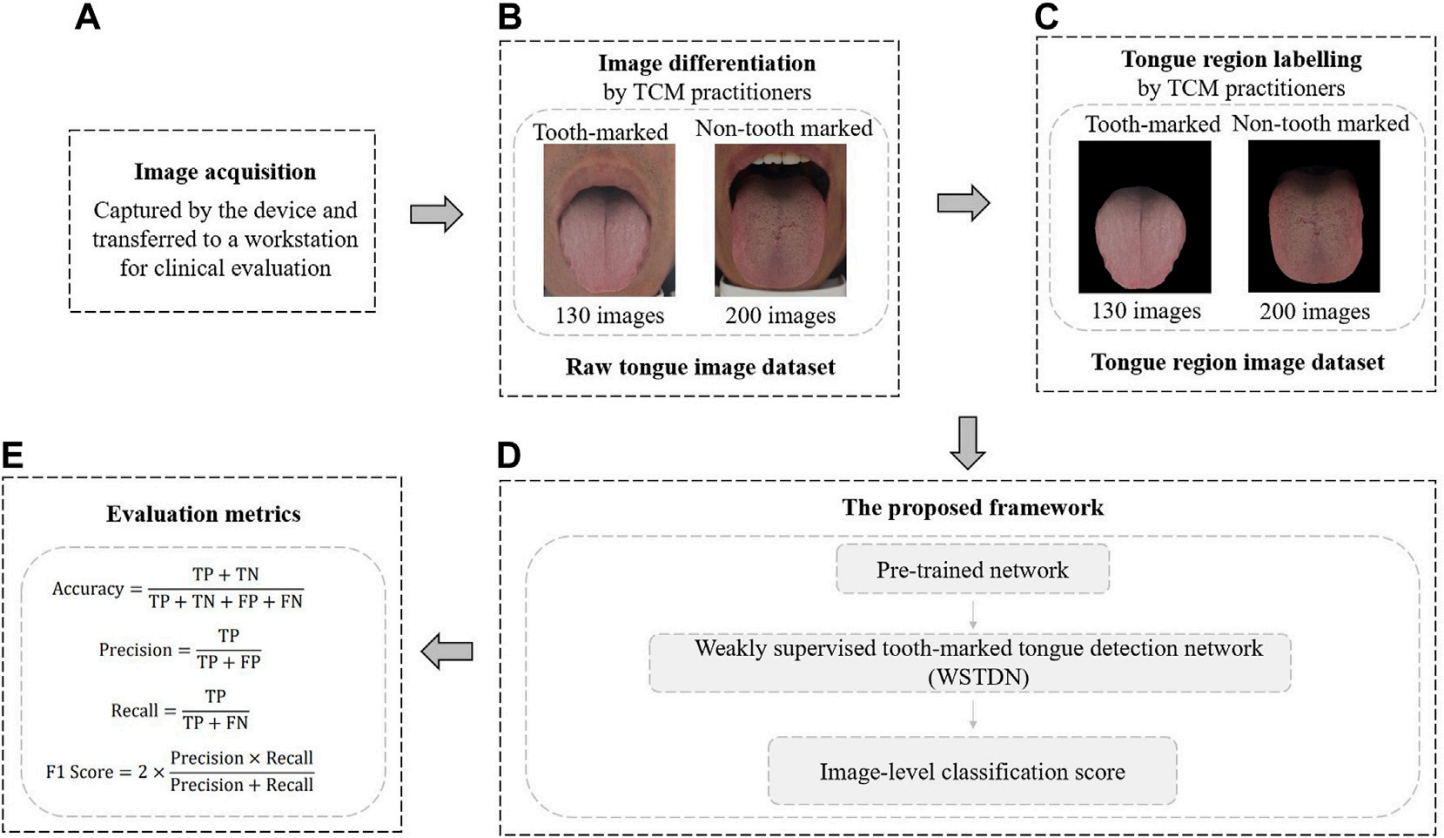
Overview of the construction of the dataset and the main processing procedures of the proposed method. **(A)** Tongue images were captured with standard equipment. **(B)** Classification of tooth-marked and non-tooth-marked samples to construct the original tongue image dataset. **(C)** Tongue region was delineated to construct a tongue image dataset. **(D)** Pre-trained CNN model, fine-tuned WSTDN model with image-level labeled data, and image-level results output. **(E)** Performance with validation metrics.

The study was approved by the local ethics committee, and the patient signed the informed consent form (IRB:2019BZHYLL0101). We used standard equipment designed by Shanghai Daoshi Medical Technology Co., Ltd. (DS01-B) to obtain tongue images from patients in the local institute. Then, we transferred the images to a workstation for clinical evaluation. Three Traditional Chinese Medicine (TCM) physicians with two to 5 years clinical experience distinguished tongue images into tooth-marked tongue or non-tooth marked tongue. All professionals are well-trained and have normal vision. The TCM clinical criteria for diagnosing tooth-marked tongues are as follows: First, observe whether there are jagged tooth marks caused by teeth pressing on the tongue on both sides of the tongue; secondly, for the tongue with inconspicuous jagged tooth marks, observe the color depth of the suspected area, in which the compressed tooth scar area typically has a darker color (Tang et al., 2020). The detailed evaluation procedure of this study consists of three steps. First, professionals discussed and acknowledged the diagnostic criteria for tooth-marked tongue. Secondly, a professional classified all 330 tongue images for identifying tooth-marked tongues, and two other professionals reviewed the classification results separately. In the case of disagreement, three professionals would discuss and make a final decision. By dividing the number of inconsistent samples reviewed by experts by the total number of samples, the inconsistency rate among experts is 14.8%. The main reasons for the inconsistency are the shadows caused by the influence of the light and the inconspicuous tooth marks on the tongue. However, in the second judgment after the discussion, opinions often reach an agreement. Let experts generate inconsistent samples, which are difficult samples. Removing these difficult samples from the training data reduces the generalization performance of the model because it only recognizes samples with obvious tooth marks. The data set after clinical screening contains 130 tooth-marked tongue images and 200 non-tooth marked images. It should be noted that in this study, the clinic only needs to provide image-level labels for the tongue image samples as tooth-marked or non-tooth marked tongues, and there is no need to provide the specific location and bounding boxes annotations of tooth-marks on the tongue. Figure 2 shows an overall pipeline for the proposed method.

### 2.2 Data Preprocessing

First, we used the Labelme software (http://labelme.csail.mit.edu/Release3.0/) to outline the tongue area, and then performed the AND operation on this area with the original image to extract the image of the entire tongue area. The purpose of extracting the tongue region was to shield the irrelevant face and the interference of the surrounding background of the tongue, so as not to affect the recognition performance of the model. The delineated tongue images were resized to 224 × 224 before entering the network, which were used in training the deep neural network. We adopted a data augmentation method of random horizontal inversion, random rotation of 0–15°, and random vertical inversion for the tongue image in order to obtain more training data for training the deep neural network.

### 2.3 The Proposed Framework

**FIGURE 3:**
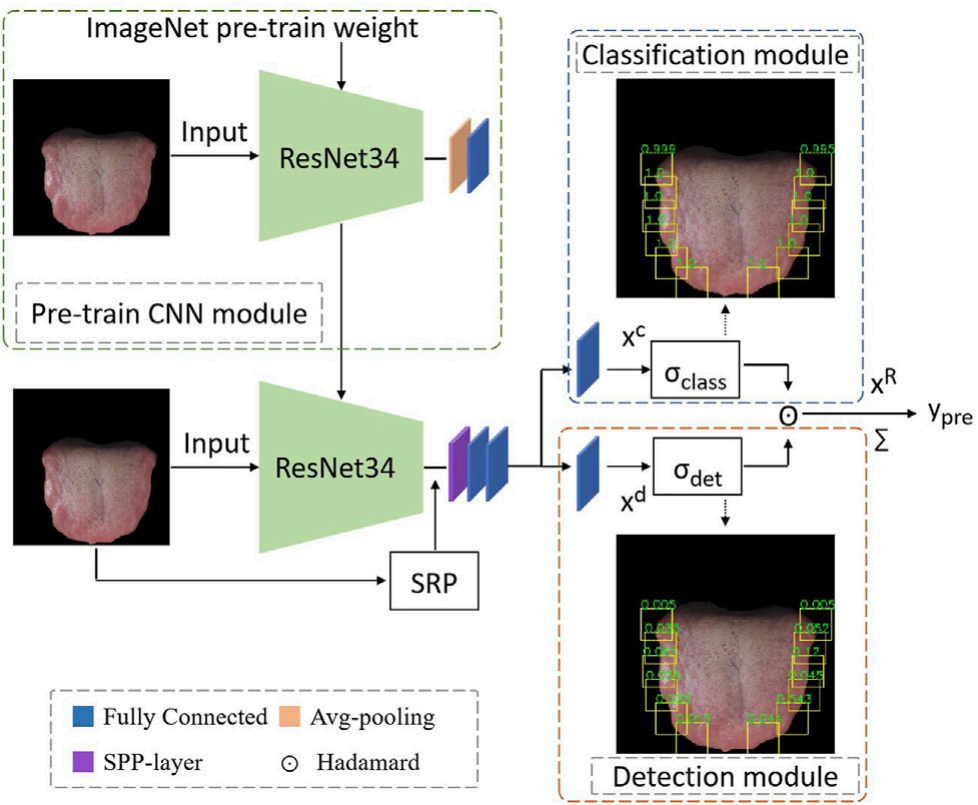
Framework of the proposed method. It includes a pre-trained CNN module; an SRP module for generating tooth mark candidate regions; an SPP-layer for obtaining and normalizing the deep features of tooth mark candidate regions; two weakly supervised branches, a classification module and a detection module; The hadamard operation of the branch results and summation yields the image-level classification results.

The proposed method mainly includes three stages, as shown in Figure 3. First, in the pre-trained CNN module, we pre-train a CNN model with the weight initialization of ImageNet using image-level annotations to distinguish between tooth-marked and non-tooth marked tongues. Subsequently, we propose the WSTDN that uses the pre-trained CNN model as the backbone and add the spatial region proposal (SRP), spatial pyramid pool (SPP), Classification module, and Detection module to achieve the weakly supervised tooth-marked tongue recognition. Finally, we fine-tune the WSTDN with only the image-level annotations, simultaneously realizing the classification of the tooth-marked tongue and the positioning of the tooth-mark area. Each module will be introduced in the following subsections.

#### 2.3.1 Pre-Trained Network

Our study is based on the premise that pre-trained CNN can be well generalized to a large number of tasks, as there is evidence that CNNs trained for image classification can bring proxies to object detection (Zhou et al., 2015). It is worth noting that these concepts are obtained implicitly without providing the network with information about the location of these structures in the image. Correspondingly, the CNN trained in tongue image classification may already implicitly contain most of the information needed to perform tooth-marked area detection. Therefore, we propose to train a CNN with the training data of tongue images and only image-level supervision (no bounding box annotations) for further tooth-marks detection. Note that the CNN has been pre-trained on ImageNet ILSVRC 2012 data (Russakovsky et al., 2015). In this study, we use the ResNet34 network (He et al., 2016), which is consistent with the previous study that has proved that ResNet34 is superior to other typical CNN models in tongue image classification (Wang et al., 2020).

**FIGURE 4:**
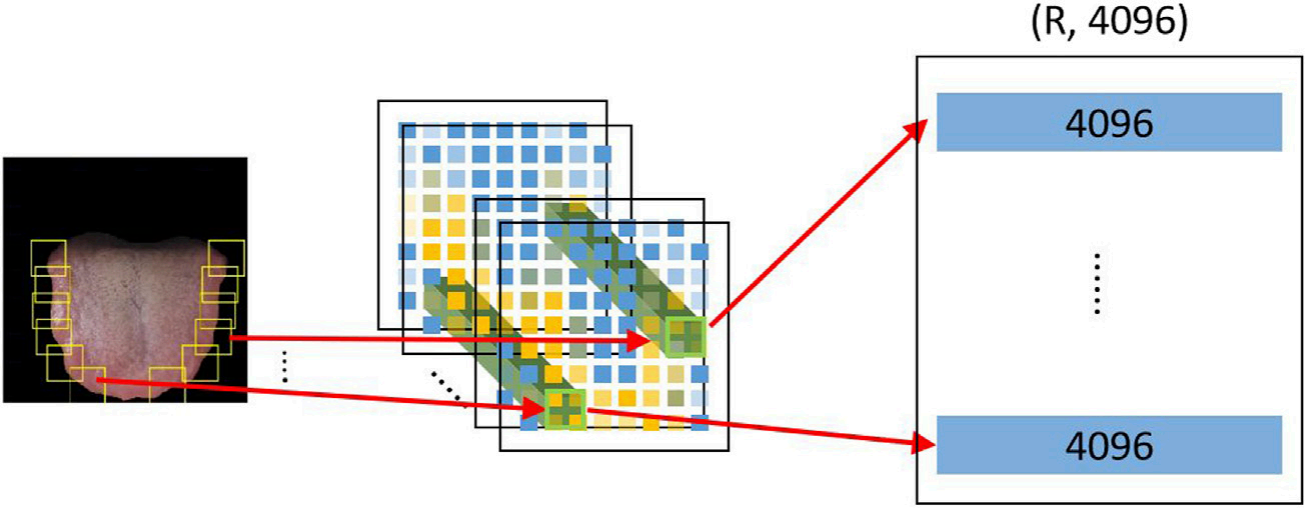
SPP layer. The green cuboid represents the deep feature of the tooth mark candidate area, and R represents the number of tooth mark candidate areas. It transforms the (h, w, 512) features of the R tooth mark candidate regions into a unified (R, 4096) size, h and w separately represent the height and width of the tooth mark candidate region.

As shown in Figure 4, the structure of ResNet34 is shown in the green dotted box, in which the two convolution layers are a group, and the residual calculation is conducted in the shortcut connection block as shown in the red dotted box. The size of tongue images and deep features are expressed as (batch size, width, height, and channel). The solid line residual arrow indicates that the input and output have the same dimension, and the dotted line residual arrow indicates that the input and output have different dimensions. Solid line ⊕ is calculated as y=f(x)+X , and the dotted line ⊕ is calculated as y=f(x)+Wx , where f(x) represents the feature calculated by the yellow matrix, and W is the convolution operation, which is used to adjust the channel dimension of X. ReLU (Nair and Hinton, 2010) was used as the activation function. The pre-trained ResNet34 model will be used as the backbone to build the proposed WSTDN for the weakly supervised tooth-marked tongue recognition in the following section.

#### 2.3.2 Weakly Supervised Tooth-Marked Tongue Detection Network

**FIGURE 5:**
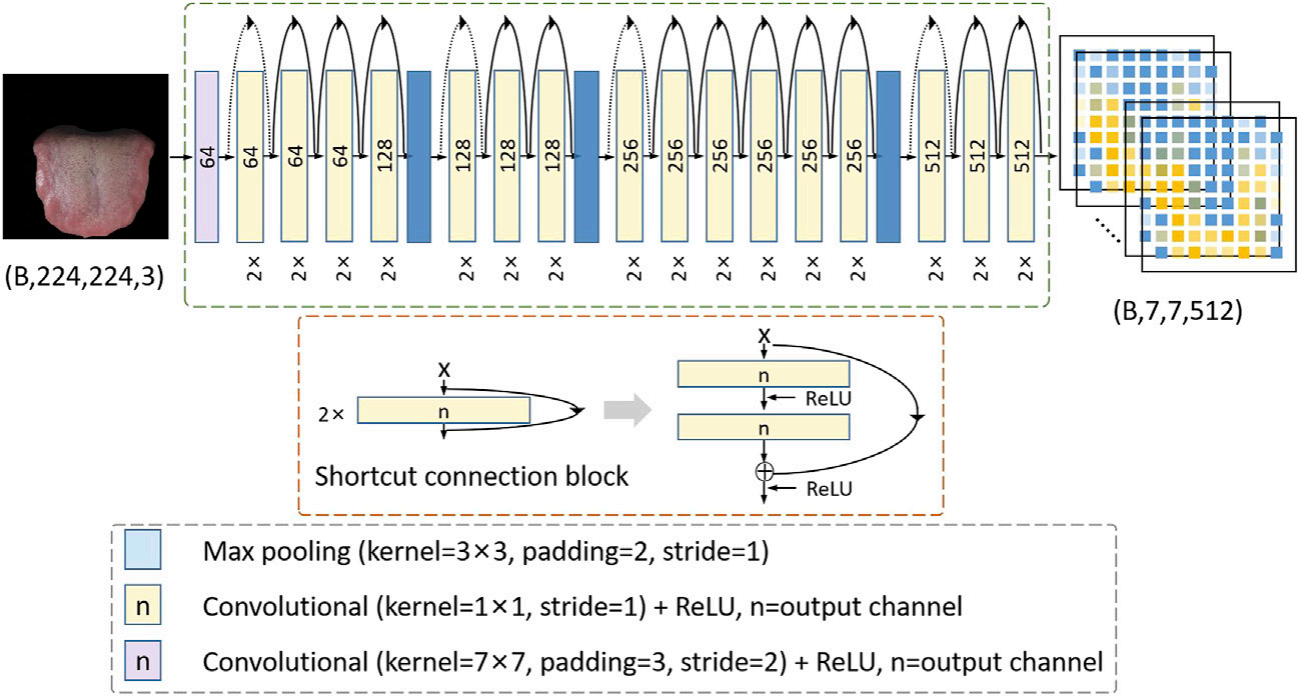
Architecture of the ResNet34 model. The size of tongue images and deep features are expressed as (batch size, width, height and channel). The solid line residual arrow indicates that the input and output have the same dimension, and the dotted line residual arrow indicates that the input and output have different dimensions.

In order to achieve the objective of weakly supervised tooth-marked tongue recognition, we have made certain improvements based on the pre-trained ResNet34 model. First, we removed the avgpool layer and fc layer behind the last BN layer in ResNet34 (that is, the classifier layer, which is only used for feature extraction), and replaced it with a spatial pyramid pool (SPP) (He et al., 2014). We implemented SPP as a network layer (Girshick, 2015) to allow the system to be trained end-to-end and improve efficiency. By introducing SPP as the network layer, we only need the original tongue image to pass through the CNN network, and we can get a deep feature of (batchsize, 7, 7, 512). As shown in the following Figure 5 of the SPP layer, the candidate area is mapped to find the corresponding candidate feature area on the 7 × 7 feature map. If the size of the candidate area is 32 × 32, from the tongue image to the deep feature, a candidate area takes at least 1 × 1 grid feature and at most 2 × 2 grid features. The SPP network layer stretches candidate feature regions of different sizes to the same size, and then inputs them to the fully connected layer, so that feature maps are calculated first, and the results of the feature maps can be shared when each candidate region is represented, saving a lot of calculation time. At this time, in the network structure, the regional-level features are further processed by two fully connected layers, and each layer contains a linear map and an activation function ReLU. Inspired by the previous research on the weakly supervised detection network (Bilen and Vedaldi, 2016), we branched out from the output of the last layer of the SPP layer into two modules, a classification module and a detection module.

#### 2.3.3 Candidate Region Generation Method Based on Location Information (Spatial Region Proposal, SRP)

The selection of tooth-marked candidate area is of great significance in tooth-marked tongue recognition. In order to generate candidate regions to use with our proposed network, we propose a novel method to select candidate areas with simple equidistant frames on both sides of the tongue. The proposed method comes from the doctor’s clinical observation. When the doctor judges whether it is a tooth-marked tongue, the main focus is on the areas of both sides of the tongue. This method avoids the large-area overlap of the candidate areas, and it is also simple and efficient to unify the selection of the candidate area of the tooth-marked tongue and the non-tooth marked tongue. h candidate region is represented, saving a lot of calculation time.

**FIGURE 6:**
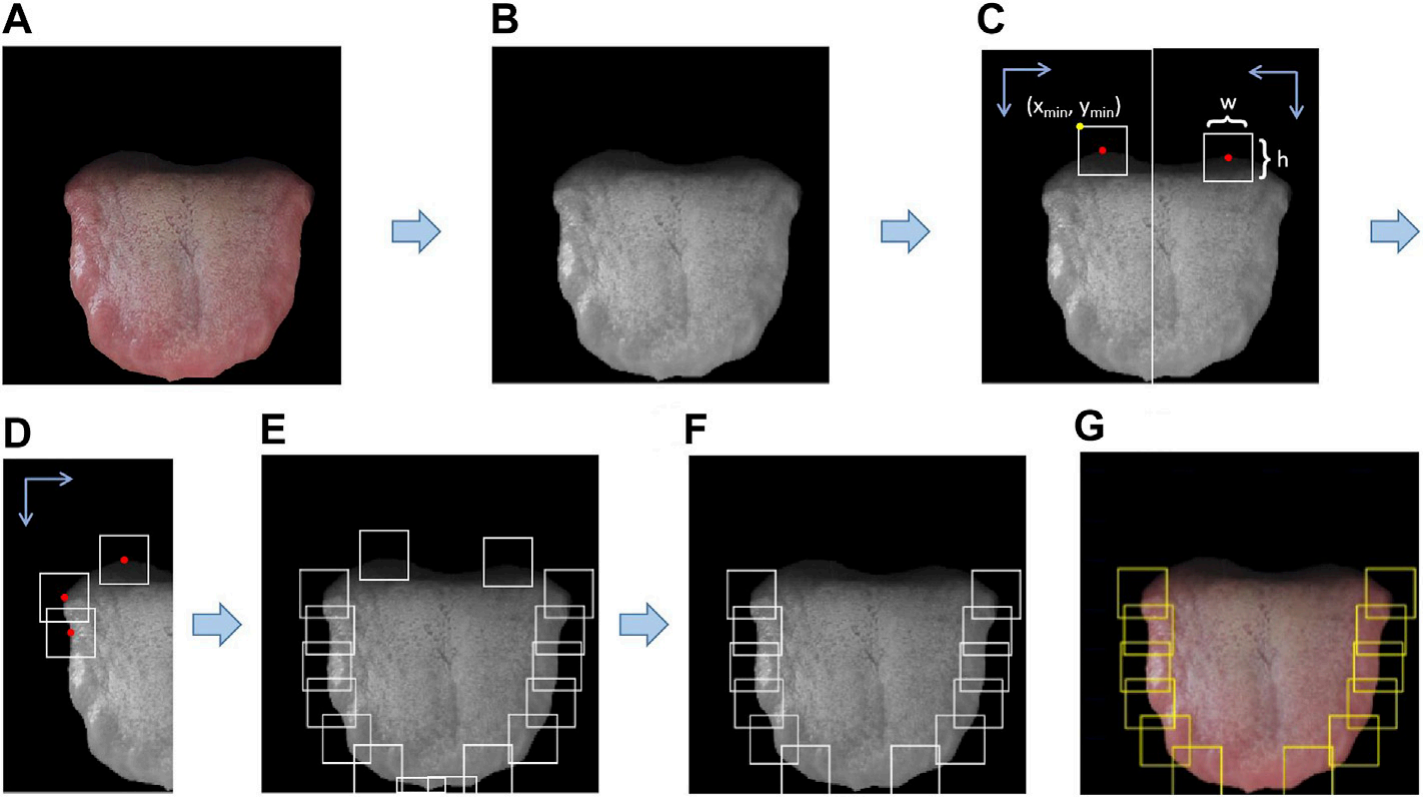
Candidate region generation method based on location information. The arrow indicates the traversal direction. Take the left as an example, from top to bottom, from left to right, and the right side is the same symmetrical operation. The red point is the first non-zero value point traversed, as the midpoint of the tooth mark candidate area, (x_min_, y_min_) is the upper left corner of the tooth mark candidate area, h and w represent the tooth mark candidate area respectively height and width.

Figure 6 showed the process of candidate region generation method based on location information. First, we convert the tongue image (Figure 6A) into a grayscale image (Figure 6B), because we only need position information, so we do not need to consider color information. Converting into a grayscale image can greatly reduce our candidate region generation time. We fall from the top to the left side of the tongue, traverse from left to middle, and get the first non-zero point, which is recorded as the midpoint. We save the minimum x, minimum y, width, and height of the candidate region according to the midpoint (Figure 6C). Then, we continue to traverse downward at equal intervals. This interval is set to 2/3 of the size of the candidate area, and the tongue is generally curved. Such a curvature can also give our candidate frame a certain horizontal displacement (Figure 6D). The right side of the tongue is from top to bottom, from right to middle, using the same method to select candidate regions. We removed the first and last candidate regions on the left and right sides (Figures 6E,F), because these two candidate regions are generally the base and tip of the tongue, which are not in the range of the tooth-marked tongue detection area. Finally, we obtain the candidate tooth-marked area on the color tongue image (Figure 6G).

#### 2.3.4 Classification Module

As shown in the Classification module in Figure 3, in order to discriminate the tooth-marked category of each candidate area, we make a linear mapping to the classification branch, and the output of this mapping is the category number *C*. Its definition is as follows (Eq. 1):

$$\left[\sigma_{class}\left(x^{c}\right)\right]ij=\frac{e^{x_{ij}^{C}}}{\sum_{k=1}^{C}e^{x_{kj}^{C}}}$$ (1)

where $x^{C}\in R^{C\times \left|R\right|}$ is the predicted scores on all classes in a certain area. Specifically, we calculate the index sum of C categories of the same region box, and then divide the current element by the value (in our case, the number of categories C=2 . Conducting the corresponding softmax transformation on the data in each column of $x^{c}$ , which is equivalent to calculate the probability of tooth marks or non-tooth marks in a certain area.

#### 2.3.5 Detection Module

As shown in the Detection module in Figure 3, in order to obtain the scores of a certain class in all candidate regions, we make a linear mapping to the detection branch, and the mapping output is also the number of classes C . Its definition is as follows Eq. 2:

$$\left[\sigma_{det}\left(x^{d}\right)\right]ij=\frac{e^{x_{ij}^{d}}}{\sum_{k=1}^{R}e^{x_{ik}^{d}}}$$ (2)

where $x^{d}\in R^{C\times \left|R\right|}$ is the predicted scores on all regions of a certain category. Specifically, for the same category, we calculate the score of the current element relative to different region boxes. Corresponding softmax transformation is performed on the data of each row of $x^{d}$ , which is equivalent to the score probability of a certain class in all regions.

#### 2.3.6 Image-Level Classification Score

Since there is no real tooth-marked area and instance-level category labels for supervision, in the two branches of our model, the classification module predicts the tooth-marked category in a certain area, and the detection module selects which areas are more likely to contain the tooth-marked area. Therefore, the final score for each area is obtained by taking the product of the two score matrices (Hadamard) ${\text{x}}_{\text{cr}}^{\text{R}}={\text{σ}}_{\text{class}}\left({\text{x}}^{\text{c}}\right)\times {\text{σ}}_{\text{det}}\left({\text{x}}^{\text{d}}\right)$ . The score $x^{R}$ is summed to obtain the final classification score y_c_, which can be defined as follows Eq. 3:

$$y_{c}=\overset{\left|R\right|}{\underset{r=1}{\sum }}x_{cr}^{R}\;$$ (3)

It is worth noting that $y_{c}$ is the sum of the product of the elements of the softmax standardized value of the area |R|, so it is in the range of (0, 1). Finally, we use cross-entropy loss to calculate the loss of our predicted $y_{c}$ and the original image-level label.

### 2.4 Implementation

The proposed weakly supervised tooth-marked tongue detection model was implemented using “PyTorch” (pytorch.org) and the Adam algorithm was used to minimize the objective function. Data augmentation was implemented with torchvision and image augmentation (github.com/aleju/imgaug). We used a NVIDIA TITAN RTX graphics card with 24G memory. The initialization of the learning rate was set to 1e-4 and the weight decay was set to 1e-4, batchsize was set to 32. The performance metrics of the computer are as follows: CPU is Intel(R) Xeon(R) Gold 5118. RAM is 64.0 GB. GPU is NVIDIA TITAN RTX. Since the computation time per image is too short and inconsistent, we selected 20 tongue images, in which there are 10 tooth-marked images and 10 non-tooth-marked images, and the total time for generating the tooth mark candidate area is calculated to obtain the time of each picture. To alleviate the problem of overfitting, if the validation accuracy did not increase for 10 epoch, an early stop was used to stop the optimization and save the model weight. Basic implementation code of the work can be available in GitHub, https://github.com/Lsx0802/WSTMD.

## 3 Experimental Results

### 3.1 Experimental Settings, Evaluation Metrics, and Comparison Methods

We divided 330 tongue images into a training set and a validation set, using 5 times four folded cross validation. The performance of the model is evaluated by calculating the average value and variance of the evaluation metrics. The experimental results are evaluated by the following four metrics: (Eq. 4) Accuracy, (Eq. 5) Precision, (Eq. 6) Recall, (Eq. 7) F1 score.

$$\text{Accuracy}=\frac{\text{TP}+\text{TN}}{\text{TP}+\text{TN}+\text{FP}+\text{FN}}$$ (4)

$$\text{Precision}=\frac{\text{TP}}{\text{TP}+\text{FP}}\;$$ (5)

$$\text{Recall}=\frac{\text{TP}}{\text{TP}+\text{FN}}\;$$ (6)

$$\text{F}1\text{ Score}=2\times \frac{\text{Precision}\times \text{Recall}}{\text{Precision}+\text{Recall}}\;$$ (7)

where TP, FP, TN, FN represent true positive, false positive, true negative, and false negative, respectively. Accuracy is the proportion of the sum of positive and negative cases correctly classified to all samples. Precision is the proportion of positive cases correctly classified to all positive cases predicted by the model. Recall is the proportion of positive cases correctly predicted by the model to all positive samples. F1 score is the balance index used to measure the accuracy of the classification model, which takes into account both precision and recall of the model and can be regarded as a harmonic average of model precision and recall. When the cost of false negative (FN) is very high (the consequences are very serious), and it needs to reduce FN as much as possible, so as to improve the recall index. Clinically, patients with tooth-marked tongues should be recognized for further treatment, so we want the model to have a higher recall value under similar accuracy conditions.

In addition, we output the candidate boxes whose scores of the model’s candidate box σ_det_ are greater than (1/the number of candidate boxes) for visual observation. Finally, we compared the proposed method with the tooth-marked tongue recognition model using multi-instance SVM (Li et al., 2019) and end-to-end convolutional network (Wang et al., 2020). We also conducted different candidate region generation methods (Uijlings et al., 2013; Li et al., 2019) for comparison.

### 3.2 Performance Comparison of Different Methods

As tabulated in Table 1, the method of (Wang et al., 2020) is directly based on ResNet34 network and tongue images for classification, and the distinguishing accuracy can reach 72.28%. After the weights initialization of ImageNet for the method of image (IPW), the accuracy can reach 79.40%. The method of (Li et al., 2019) can achieve the accuracy of 90.34% by extracting instance and using ResNet34 to directly classify. Using ResNet34 to extract deep features followed by using MiSVM classification in the method of instance MiSVM (Li et al., 2019) can further improve the performance, reaching 93.49%. Compared with the method of (Wang et al., 2020), the proposed method, and (Li et al., 2019) have achieved a large performance improvement, which may be due to our further extraction of the tongue tooth-marked informative area.

**TABLE 1:** Performance comparison of different methods for tooth-marked tongue recognition.

Backbone	Methods	Accuracy	Precision	Recall	F1-score
Resnet34	Image (Wang et al., 2020)	0.7228 ± 0.0147	0.7344 ± 0.1025	0.5091 ± 0.1181	0.5863 ± 0.0678
	Image (IPW) (Wang et al., 2020)	0.7940 ± 0.0442	0.8188 ± 0.8000	0.6275 ± 0.1574	0.6961 ± 0.1020
	Instance (Li et al., 2019)	0.9034 ± 0.0227	0.9185 ± 0.0344	0.8294 ± 0.3922	0.8711 ± 0.0298
	instance_MiSVM (Li et al., 2019)	0.9349 ± 0.0255	0.9332 ± 0.0349	0.9002 ± 0.0550	0.9156 ± 0.0340
	WSTDN	0.9197 ± 0.0759	0.8745 ± 0.1087	0.9427 ± 0.1197	0.9026 ± 0.0954

However, compared with (Li et al., 2019) using MiSVM classification, our performance is not better than it. The reason may be that after the instance is generated, they have performed manual screening to achieve higher performance. On the other hand, compared to the softmax classifier in the proposed end-to-end network, the classification performance of SVM may be better than using softmax classification in the absence of training data (Tang, 2013; Girshick, 2015). However, (Li et al., 2019) with SVM is not an end-to-end network, while the proposed method of optimizing the softmax classifier can simplify the training and test process (Girshick, 2015).

### 3.3 Performance Assessment of Candidate Region Proposal

For the method of generating candidate regions, we comparatively experimented with selective search (Uijlings et al., 2013), edges box (Zitnick and Dollár, 2014), convex defect detection (Li et al., 2019), and our method SRP. The definition of a candidate box is that if the number of edge contours that are completely contained in a box, then the target has a high probability in this box (Zitnick and Dollár, 2014). However, under the condition of tooth-marked tongue classification, it is difficult to frame the tooth-marked area with the method of (Zitnick and Dollár, 2014), because the tooth-marked area and the tongue are connected. The proposed method and (Li et al., 2019) both use the prior knowledge of tooth marks. According to observations, the tooth-marked tongue does have convex defects on the edge of the tongue. Convexity detection can be used to frame the area, but the non-tooth marked tongue convexity area is not obvious, and it is difficult to achieve the unity of tooth-marked and non-tooth marked tongues.

**FIGURE 7:**
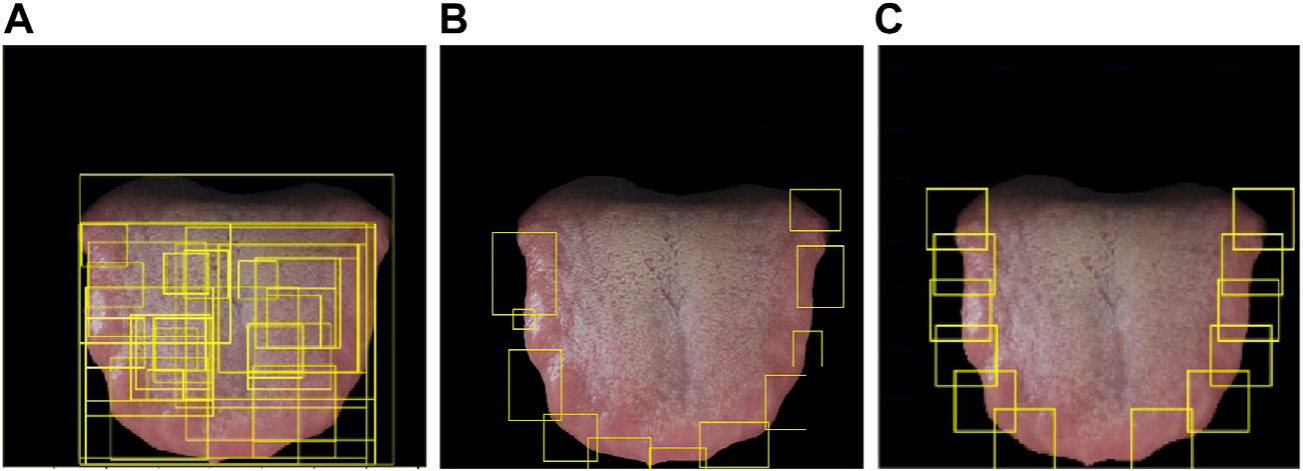
Generation of different candidate region proposal methods. **(A)** Selective search (Uijlings et al., 2013), **(B)** convex defect detection (Li et al., 2019), **(C)** SPR (ours). Note that the yellow box is the candidate box of the tooth mark region generated by different methods.

Figure 7 shows the comparison of candidate regions of different candidate region generation methods. The method (Uijlings et al., 2013) in Figure 7A can generate a large number of candidate frames, but there are many invalid candidate regions, and the candidate regions overlap seriously. The method (Li et al., 2019) in Figure 7B is very good in selecting the tooth-marked candidate areas, but the convex defect of the tongue tip will be detected, and the part of the tongue tip is not the informative area for the identification of the tooth mark, so it needs to be manually screened and removed later. In our method in Figure 7C, it can efficiently select the informative area for the identification of tooth marks.

From the comparison of the results in Table 2, we can find that our SPR method is better than the method of (Uijlings et al., 2013). It may be because our method selects the information area for identification of tooth marks, rather than invalid areas such as the tip of the tongue and the base of the tongue, and our method does not have the large-area overlap of the candidate frames in (Uijlings et al., 2013). In addition, the time consuming of SRP is much less than the method in (Uijlings et al., 2013), probably because they use the color information of the three channels of RGB, while our method uses Gray-scale image, and the traversal method on the left and right sides reduces a lot of traversal time.

**TABLE 2:** Performance comparison of different region proposal methods.

Backbone	Methods	Accuracy	Precision	Recall	F1-score	Time per image
WSTMD	Selective search (Uijlings et al., 2013)	0.8708 ± 0.0979	0.8522 ± 0.1215	0.8068 ± 0.1495	0.8300 ± 0.1312	0.30
	SPR (ours)	0.9197 ± 0.0759	0.8745 ± 0.1087	0.9427 ± 0.1197	0.9026 ± 0.0954	0.19

### 3.4 Ablation Study

As shown in Table 3, IW represents using ImageNet weights to initialize the WSTDN model, and TL means using transfer learning to directly train a model for tongue image classification, and then initialize the WSTDN model with its weights. IW + TL is the method we proposed, using ImageNet weights to initialize the ResNet34 model, followed by using the tongue images and image-level labels to train the ResNet34 model, and finally using its weights to initialize the WSTMD model. It can be observed that, when we use the IW method to initialize our tooth-marked detection model, the effect is not as good as using the TL method. The TL weight is learned from the tongue image and has a certain ability to discriminate the tongue image, so this transfer learning method achieves a better effect. The reason why it is inferior to the IW + TL method may be that the weights are initialized randomly, and there is no good discrimination ability, which may lead to relatively lower performance.

**TABLE 3:** Performance of ablation study in the proposed WSTDN method.

Backbone	Methods	Accuracy	Precision	Recall	F1-score
WSTDN	IW	0.7460 ± 0.0569	0.8403 ± 0.1120	0.4795 ± 0.2397	0.5661 ± 0.1795
	TL	0.8848 ± 0.0477	0.8777 ± 0.0610	0.8254 ± 0.0778	0.8506 ± 0.0607
	IW + TL	0.9197 ± 0.0759	0.8745 ± 0.1087	0.9427 ± 0.1197	0.9026 ± 0.0954

### 3.5 Visualization

**FIGURE 8:**
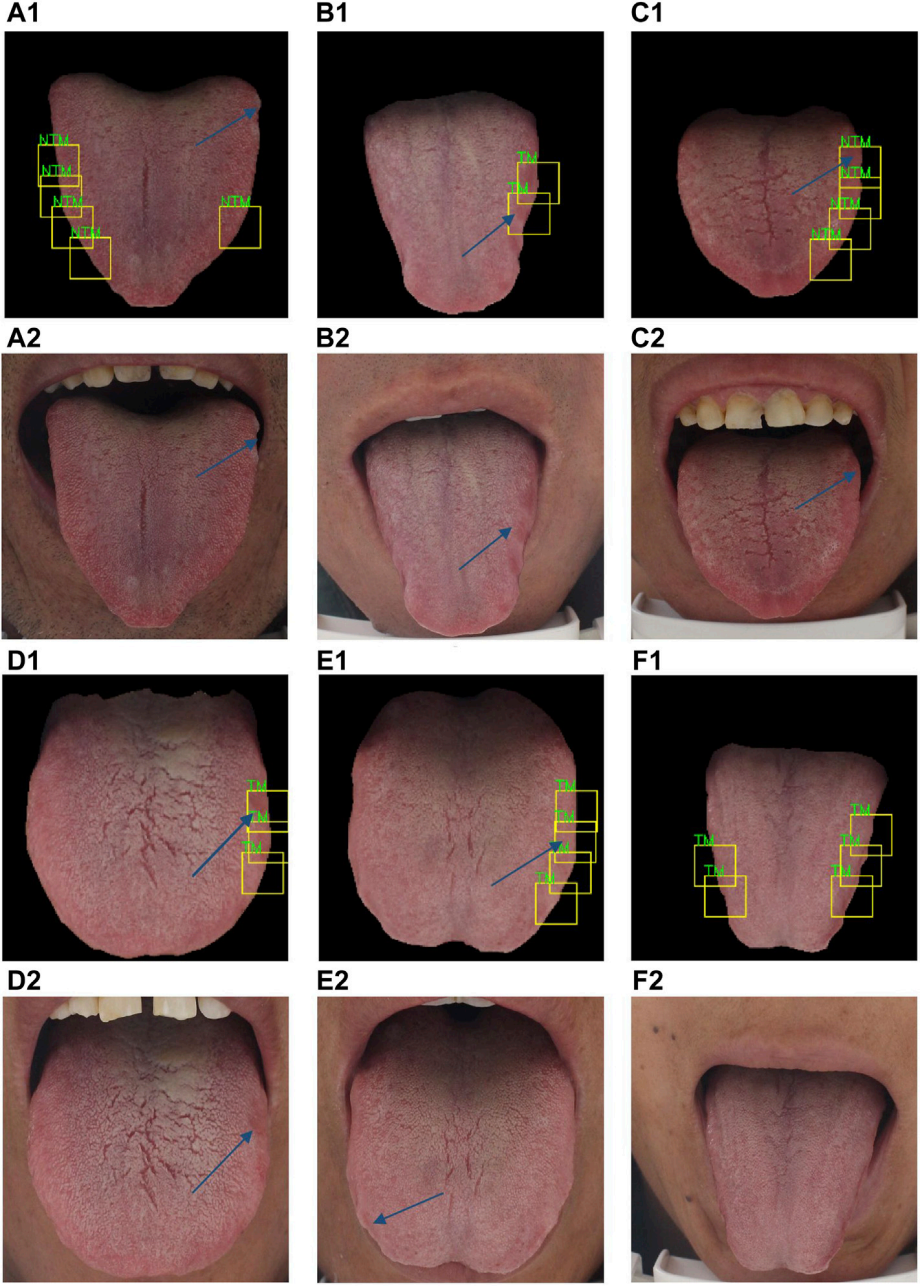
Representative cases of tooth-marked tongue recognition by the proposed method. A2, B2, C2, D2, E2, and F2 are original tongue images, while A1, B1, C1, D1, E1 and F1 are corresponding prediction results of tooth marks with bounding boxes.

Figure 8 showed some examples of tooth-marked tongue recognition by the proposed method. As shown in Figure 8(A1) and (C1), the edges on both sides of the tongue are flat without tooth marks and there are physiological defects at the root and tip of the tongues, which are not tooth marks. The proposed model avoids these physiological defect areas well, and the areas on both sides of the tongue are identified correctly. Furthermore, as shown in Figure 8(B1), the tooth-marked tongue has distinctive characteristics, including tooth marks and color changes in the tooth pressure area. The proposed model can identify them correctly. As shown in Figure 8(D1), some small color difference changes that are not easy to recognize or easily ignored by human eyes can be accurately identified by the model. The side indicated by arrow in Figure 8(E2) is more obvious than that indicated by the arrow in Figure 8(E1), but the focus area of the model is single, and only one side identification area is concerned. Even if the typical tooth mark area indicated by the arrow in Figure 8(E2) is not focused, the tooth-marked tongue is correctly identified by the identified area. In Figure 8(F1), the model recognition is incorrect. The tongue image is squeezed on the edge of the tongue due to the tension of the tongue when the patient extends the tongue. The model mistakenly recognizes it as a tooth-marked tongue. Therefore, high-quality tongue body imaging is very important for tooth-marked tongue recognition.

## 4 Discussion

The characteristics of a tongue can reflect the internal health of the body and the severity or progression of the disease in traditional Chinese medicine. Traditional Chinese medicine can distinguish the clinical symptoms and choose appropriate treatment strategies. As one of the most important tongue features, tooth-marked tongue has been used as an effective signature of health in traditional Chinese medicine. Our model may provide an important research paradigm for distinguishing tongue features, diagnosing syndromes of traditional Chinese medicine, tracking disease progression, and evaluating intervention effects, showing its unique potential in clinical applications. Potentially, the proposed method can also be used to evaluate the efficacy of the drug by detecting the tooth marks of the tongue for noninvasive ethnopharmacological evaluation (Wang et al., 2022). The pathological cause of tooth-marked tongue is the change of microcirculation of the tongue due to the compression of the tongue by the teeth. For example, there are blood supply disorders, local hypoxia, insufficient nutrition, tissue edema, etc. in the area of tooth compression, and eventually tooth marks are formed (Wang et al., 2020). Previous studies have shown that tooth-marked tongue is closely related to human health and disease. The tooth-marked tongue is related to human gender and age, in which males have fewer tooth marks and women have more tooth marks, and the relationship between the increase of age and the reduction of tooth marks is more obvious (Hsu et al., 2019). In addition, there is a positive correlation between lung capacity and tooth-marked tongue. The occurrence rate of tooth-marked tongue is higher in patients with moderate or higher abdominal force. The occurrence of tooth-marked tongue in hypertensive patients without anemia is significantly related to the increase in hematocrit. Patients with hypoalbuminemia is mostly pale with tooth-marked tongue (Jing, 2002). The number of tongue features such as tooth marks, average coverage area, maximum coverage area, minimum coverage area, and organs corresponding to the coverage area can be used as criteria for evaluating chronic kidney disease or breast cancer (Lo et al., 2013; Chen et al., 2021). Patients with subacute eczema have a higher incidence of tooth marks than patients with acute eczema and patients with chronic eczema (Yu et al., 2017).

The proposed SRP module is more in line with the observation rules of traditional Chinese medicine physicians. First, according to the results of (Sun et al., 2019; Wang et al., 2020), tooth marks exist on both sides of the tongue, and the tip of the tongue and the center of the tongue are not the main discriminating areas. We use the method of equidistant selection on both sides of the tongue, which can efficiently extract the candidate regions of tooth marks. In contrast, the method of (Li et al., 2019) based on the convexity area detection method can extract the tooth-marked candidate area on the tooth-marked tongue. However, there is little obvious concave and convex information on the non-tooth marked tongue, which makes it are difficult to generate the candidate regions of the tooth-marked and non-tooth marked tongues efficiently and uniformly. In addition, the method of (Uijlings et al., 2013) does not use the prior knowledge of tooth marks. The generated candidate areas have a large number of invalid frames and a lot of area overlap. It can be seen from Tables 2, 3 that our method has advantages in the generation time of candidate tooth-mark areas and model classification performance.

We initialize the CNN model based on ImageNet weights and use the transfer learning method to obtain better tooth-marked tongue detection results. Inspired by (Zhou et al., 2016), when they trained this scene classification convolutional network, the labels they gave were scene labels without any object calibration. The network neurons naturally evolved into object detectors. Therefore, we consider the tooth-marked tongue recognition as a tooth-marked area detection problem, rather than an instance-level classification problem. Unlike other detection methods such as (Girshick, 2015), we do not have instance-level labels. Our method is inspired by the weakly supervised deep target detection method (Bilen and Vedaldi, 2016), which uses image-level labels to classify and detect candidate regions. Based on the candidate regions, the image-level prediction results are obtained. By filtering the scores of our detection branches, we can better locate the tooth-marked area predicted by the model. By comparison, extracting candidate regions by filtering and labeling examples requires a lot of labeling costs in (Li et al., 2019).

Finally, this study still has certain shortcomings. First, the amount of data in this study is not large enough. The data comes from the same center, and the study of multi-center data has not been carried out, which will be conducted in the future. Secondly, there may be uncertainties in the gold standard label of clinical tooth-marked tongue by the TCM due to the challenging of the recognition of tooth-marks. Providing uncertainty estimates is not only important for a safe decision-making in high-risks fields, but also crucial in fields where the data sources are highly inhomogeneous and labeled data is rare (Gal, 2016). Uncertainty research (Kendall and Gal, 2017; Gawlikowski et al., 2021) will be introduced in the follow-up. In addition, the proposed method is based on the segmented tongue body to distinguish, and the deviation of the tongue body segmentation may bring discriminant bias. The follow-up will consider the construction of multi-task learning for segmentation and detection to make two tasks promote each other, thereby further improving the detection accuracy. Finally, the proposed method has not yet carried out prospective experiments. Since tooth-marked tongues are less than non-tooth marked tongues in clinical practice, there is uneven sample distribution. This is the follow-up model that needs to be considered for clinical prospective experiments.

## 5 Conclusion

In this study, we proposed a weakly supervised learning method of tooth-marked tongue recognition, by pre-training a CNN model that classifies tooth-marked tongues, and then transferring it to the WSTDN with the utilization of only image-level labels (tooth-marked tongue/non-tooth marked tongue) for fine-tuning. Experimental results demonstrate that the proposed method with only image-level label annotations is effective, and its performance is comparable to that of the deep neural network method that requires a large number of instance labels. In addition, this method uses the CNN network for end-to-end training, and the tooth-marked tongue classification is achieved while the tooth-marked areas is located, which is convenient for clinical diagnosis and interpretation. This method is expected to play an important role in the clinical diagnosis of traditional Chinese medicine, especially in noninvasive ethnopharmacological evaluation.

## Data Availability

The original contributions presented in the study are included in the article/Supplementary Material, further inquiries can be directed to the corresponding authors.
